# Total urinary polyphenols and ideal cardiovascular health metrics in Spanish adolescents enrolled in the SI Program: a cross-sectional study

**DOI:** 10.1038/s41598-022-19684-6

**Published:** 2022-09-14

**Authors:** Emily P. Laveriano-Santos, Camila Arancibia-Riveros, Isabella Parilli-Moser, Sonia L. Ramírez-Garza, Anna Tresserra-Rimbau, Ana María Ruiz-León, Ramón Estruch, Patricia Bodega, Mercedes de Miguel, Amaya de Cos-Gandoy, Vanesa Carral, Gloria Santos-Beneit, Juan M. Fernández-Alvira, Rodrigo Fernández-Jiménez, Rosa M. Lamuela-Raventós

**Affiliations:** 1 Department of Nutrition, Food Science and Gastronomy, School of Pharmacy and Food Sciences XIA, Institute of Nutrition and Food Safety (INSA-UB), University of Barcelona, 08921 Santa Coloma de Gramenet, Spain; 2 Consorcio CIBER, M.P. Fisiopatología de la Obesidad y Nutrición (CIBERObn), Instituto de Salud Carlos III (ISCIII), 28029 Madrid, Spain; 3 Department of Internal Medicine, Hospital Clínic, Institutd’Investigacions Biomèdiques August Pi I Sunyer (IDIBAPS), University of Barcelona, 08036 Barcelona, Spain; 4 Mediterranean Diet Foundation, 08021 Barcelona, Spain; 5 Foundation for Science, Health and Education (SHE), 08008 Barcelona, Spain; 6 Centro Nacional de Investigaciones Cardiovasculares (CNIC), 28029 Madrid, Spain; 7 The Zena and Michael A. Wiener Cardiovascular Institute, Icahn School of Medicine at Mount Sinai, New York, NY 10029 USA; 8 CIBER de Enfermedades Cardiovasculares (CIBERCV), 28029 Madrid, Spain; 9 Hospital Universitario Clínico San Carlos, 28040 Madrid, Spain

**Keywords:** Biomarkers, Cardiology, Medical research, Risk factors

## Abstract

To study the relationship between urinary total polyphenol excretion (TPE) in adolescents and ideal cardiovascular (CVH) metrics. 1151 adolescents aged 12.04 (0.46) years participating in the SI! Program for Secondary Schools were selected based on the availability of urine samples and information required to assess CVH metrics. Data on health behaviours (smoking status, body mass index, physical activity, and healthy diet) and health factors (blood pressure, total cholesterol, and blood glucose) were used to calculate the CVH metrics. TPE in urine was analysed by a Folin-Ciocalteu method after solid-phase extraction. Associations between TPE (categorized into tertiles) and CVH metrics (total and separate scores) were assessed using multilevel mixed-effect regression models. Higher TPE levels were associated with higher (healthier) CVH scores and ideal smoking status (OR 1.54, 95% CI 1.10; 1.87, *p* value = 0.007), physical activity (OR 1.12, 95% CI 1.02; 1.23, *p* value = 0.022) and total cholesterol (OR 1.78, 95% CI 1.16; 2.73, *p* value = 0.009) after multivariate adjustment. An association between TPE and total CVH scores was observed only in boys. Girls with higher TPE had higher rates of ideal total cholesterol and blood pressure. According to our findings, higher urinary TPE is related to better CVH scores, with relevant differences in this association by gender.

## Introduction

Although the clinical events of cardiovascular disease (CVD) generally occur in adulthood, cardiovascular risk factors develop in childhood and adolescence due to the accumulation of unhealthy behaviours^1^. In order to promote and monitor cardiovascular health (CVH), the American Heart Association defined the concept of ideal CVH based on seven metrics (smoking status, body mass index, physical activity, diet, blood pressure, total cholesterol, and blood glucose)^2^.

Following a Mediterranean diet is known to help prevent CVD, partly because it includes polyphenol-rich foods^3,4^. Cardiovascular protection of polyphenols could be due to their anti-inflammatory and antioxidant properties, having an effect on endothelial function, oxidative stress, and the metabolism of glucose and lipids^5–7^. Besides, food frequency questionnaires, dietary history interviews, or 24-h diet recalls are the most common dietary assessment tools to estimate dietary polyphenol intake^8^. However, in recent years the measurement of urinary total polyphenols (TPE) has been considered a reliable biomarker of total polyphenol intake^9^. In a recent study on Spanish adolescents from the SI! Program, we found that higher polyphenol excretion in urine was associated with lower values of body fat percentage, triglycerides (TG), total cholesterol, and low-density lipoprotein cholesterol (LDL-c) in boys, and lower blood pressure in girls^10^, all of which are well-known cardiovascular risk factors. Additionally, previous studies have reported a correlation between CVH metrics in adolescence with subclinical markers of CVD in adulthood^11,12^. Therefore, to shed light on the effect of polyphenol intake on CVH at an early stage of life, the present study investigated the relationship between urinary polyphenol levels and CVH metrics in adolescents.

## Methods

### Study design and participants

This is a sub-study carried out based on data from the SI! (*Salud Integral-Comprehensive Health*) Program for Secondary Schools is a cluster-randomized controlled intervention trial (NCT03504059) aiming to evaluate the effectiveness of an educational program to improve CVH in adolescents. It was conducted from 2017 to 2021 in 1326 participants from 24 Spanish secondary public schools. A detailed description of the study design and recruitment procedures is available elsewhere^13^. The study protocol was approved by the Joint Commission on Ethics of the Instituto de Salud Carlos III in Madrid (CEI PI 35_2016), the Fundació Unió Catalana d’Hospitals (CEI 16/41), and the University of Barcelona (IRB00003099) and carried out in accordance with the Helsinki Declaration. Parents or caregivers provided assent and written informed consent at the beginning of the study.

For this cross-sectional study, baseline data of 1151 adolescents (47% girls) enrolled in the SI! Program were used. Participants with unavailable urine samples (n = 13), diagnosed with diabetes (n = 6) or hypertension (n = 1), that had taken any drugs or supplements (n = 116) the day prior to the data collection, and with missing data for any of the CVH metrics (n = 39) were excluded.

### Quantification of total polyphenol excretion (TPE) in urine samples

A validated Folin–Ciocalteu spectrophotometric method described by Medina-Remón et al*.* was used to determine TPE levels in spot urine samples^9^. A prior solid phase extraction was carried out using OASIS 30 mg MAX 96 well plates (Waters, Milford, MA) to remove potential interferences with the Folin–Ciocalteu reagent^9^. Gallic acid (GA) (Sigma-Aldrich, St. Louis, MO, USA) was used as a reference for TPE quantification, and its calibration curve ranged from 0.7 to 16 mg/L. Creatinine was measured using the Jaffé alkaline picrate method adapted for thermo microtiter 96-well plates by Medina-Remón et al*.*^9^ A calibration curve for creatinine was prepared with a standard (Fluka, St. Louis, MO, USA) at values from 0.5 to 1 mg/L. The coefficient of variation between measures of GA and creatinine was less than 15%. Finally, TPE was normalized by creatinine, expressed as mg GA equivalent/g creatinine and categorized into tertiles.

### Cardiovascular health assessment

Seven CVH metrics were calculated in the adolescents using the cut-off values stipulated by the American Health Association, as summarized in Table 1, comprising four health behaviours and three health factors^2^*.*

**Table 1:** Cardiovascular health metrics as defined by the American Health Association.

CVH Component	Ideal metric	Non-ideal metric
**Health behaviours**		
Smoking status	Never smoked a whole cigarette	All other individuals
Body mass index	< 85th percentile	≥ 85th percentile
Physical activity	≥ 60 min/day MVPA every day	< 60 min/day MVPA or no physical activity every day
Healthy diet score	4 components^a^	0–3 components^a^
**Health factors**		
Total cholesterol	< 170 mg/dL	≥ 170 mg/dL
Blood glucose	< 100 mg/dL	≥ 100 mg/dL
Blood pressure	< 90th percentile	≥ 90th percentile

CVH, cardiovascular health; MVPA, moderate-to-vigorous physical activity.

^a^Diet score is based on the following dietary recommendations: fruits and vegetables ≥ 4.5 servings/day, fish ≥ two 3.5-oz servings/week, fibre-rich whole grains ≥ 3 servings/day, and sugar-sweetened beverages ≤ 450 kcal (36 oz)/week, all scaled to a diet of 2000 kcal/day.

### Health Behaviours

Smoking status was evaluated by a confidential self-reported questionnaire^13^ and was considered ideal when the participant had never smoked a whole cigarette.

Weight was measured using an electronic scale (OMRON BF511, OMRON HEALTHCARE Co., Muko, Kyoto, Japan) and height by a portable stadiometer (SECA 213, Hamburg, Germany) while participants wore light clothing and no shoes. Both measurements were conducted by a trained staff^13^. The body mass index (BMI) was calculated as weight in kilograms divided by height in meters squared (kg/m^2^). BMI z-scores and percentiles were calculated based on the median values in adolescents by age and gender according to the Center for Disease Control (CDC)^14^. BMI was considered ideal when values were under the 85th percentile.

Moderate-to-vigorous physical activity (MVPA) was measured with an accelerometer (ACTIGRAPH WGT3X-BT, ActiGraph, Pensacola, USA) worn on the non-dominant wrist for seven consecutive days and applying the cut-points of Chandler et al*.*^15^. In participants with missing accelerometer data, we used information reported from a validated questionnaire^13,16^, estimating the MVPA according to the frequency and duration of recreational physical activity and competitive sports done inside or outside schools, on schooldays and at weekends. A conversion factor was used to calculate MVPA in terms of minutes per day according to the questionnaire. Participants with ≥ 60 min/day of MVPA were considered to have an ideal level of physical activity.

Regarding diet, information about the intake of fruits, vegetables, fish, fibre-rich whole grains, and sugar-sweetened beverages was obtained using a validated 157-item semi-quantitative food frequency questionnaire (FFQ) filled out by the families^17,18^. A healthy diet score was based on fruits and vegetables ≥ 4.5 servings/day, fish ≥ 2 servings/week, fibre-rich whole grains ≥ 3 servings/day, and sugar-sweetened beverages ≤ 36 oz or 1065 mL/ week based on 2000 kcal of total daily energy intake. The validated non-quantitative self-reported Children's Eating Habits Questionnaire (CEHQ), was filled out by adolescents through the face-to-face interview method conducted by trained staff^19^. It was used to evaluate dietary intake in cases without available FFQ data. In the CEHQ, the frequency of food consumption was assessed as times per month, week, or day, and categorized in eight responses: 1 = never or less than once per month, 2 = once or twice per week, 3 = four or six times per week, 4 = once per day, 5 = two or three times per day, 6 = four or six times per day, 7 = more than six times per day, 8 = unknown. A conversion factor was used to transform questionnaire answers into weekly or daily consumption frequencies. Finally, subjects who had an ideal intake of all four diet components achieved an ideal healthy diet score.

### Health factors

Total cholesterol (TC) and blood glucose (BG) levels were measured by trained staff and determined using a portable biochemical analyser (CardioChek Plus, Polymer Technology System Inc., Indianapolis, USA) in finger-prick capillary samples of whole blood (approximately 40 µL) taken early in the morning after overnight fasting^13^. In adolescents, ideal levels of TC have been defined as < 170 mg/dL and BG, < 100 mg/dL.

Blood pressure (BP) was measured when participants were in a sitting position using a digital monitor OMRON M6 (OMRON HEALTHCARE Co., Muko, Kyoto, Japan). Duplicate measurements were taken at two- or three-minute intervals after the participants relaxed^13^. Lowest BP values were used to calculate BP centiles according to gender-specific and age-specific z-scores from the High Blood Pressure Working Group of the National Blood Pressure Education Program for children and adolescents^20^. Systolic BP (SBP) and diastolic BP (DBP) were considered ideal when under the 90th percentile.

### Cardiovascular health score

The overall CVH score was calculated by assigning one point for each ideal metric (health behaviour or factor), and zero points for each non-ideal metric, being categorized as poor (0–3 points), intermediate (4–5 points), and ideal (6–7 points), as previously described^21^.

### Sociodemographic characteristics

Parental education and household income were assessed based on a self-completed questionnaire for parents or legal guardians^13^. The highest parental education level corresponded to university studies according to the International Standard Classification of Education^22^. Household income was categorized as low, medium, or high, based on the reference salary for the Spanish population^23^. Puberty development was assessed according to Tanner maturation stages based on self-reports by the participants using pictograms^24^.

### Statistical analysis

Descriptive characteristics of participants were reported for the total population and by gender, using mean and standard deviations for continuous variables due to approximate normal distribution, and frequencies with percentages for categorical variables. T-test was carried out to analyze differences between gender. Participants were classified into tertiles of TPE (T1 < 85.8 mg GAE/g creatinine, T2 85.8–140.5 mg GAE/g creatinine, and T3 > 140.5 mg GAE/g creatinine). Pearson chi-square test (X^2^) and one-way analysis of variance were used to assess the unadjusted difference in frequencies and mean across tertiles of TPE, respectively.

Multilevel mixed-effect linear regression models, with robust error variance, were used to evaluate the association between tertiles of TPE with the CVH score (continuous). The results of the regression models are expressed as unstandardized B coefficients and their 95% confidence interval (CI). In model 1, the fixed effect was gender (girls/boys); in model 2 were added age (continuous), fasting (yes/no), Tanner maturation stages (from I to V), and TG; finally, model 3 was additionally adjusted by highest parental education (yes/no), and household income (low, medium, and high). Akaike information criteria was applied to indicate the better regression model. To study the association between tertiles of TPE and each ideal CVH metric, multilevel mixed-effect logistic regression was performed using robust error variance, expressed as odds ratio (OR) and 95% CI and adjusted by the same variables considered in regression model 3. The associations of TPE with each CVH metric were analysed by comparing the highest with the lowest tertile of TPE. Municipalities (Barcelona/Madrid) and schools were included as a random effect. We evaluated the potential modifying effect of gender on the association between tertiles of TPE and CVH in an interaction analysis using cross-product terms between TPE and gender in the analysis. This analysis was also stratified by gender to evaluate potential modification. Linear trends were assessed using orthogonal polynomial contrasts. All statistical analyses were carried out using STATA statistical software package version 16.0 (StataCorp, College Station, TX, USA), and R 4.1.1 (R Foundation for Statistical Computing, Vienna, Austria). Statistical tests were two-sided, and *p* values under 0.05 were considered significant.

## Results

### Characteristics of the study population

The characteristics of the study participants are summarized in Table 2. Over half of the adolescents were boys (53%), and the mean age was 12.04 (0.46) years. Boys had higher SBP and BG levels, whereas girls had higher DBP and triglyceride levels. A higher intake of fruits and vegetables, as well as a higher percentage of lower MVPA (< 60 min/day), was observed in girls. No significant differences in sociodemographic variables were observed by gender (Table 2).

**Table 2:** Baseline characteristics of the SI! Program cohort at baseline by gender.

	N	Boys (n = 607)	Girls (n = 544)	*p* value
Age (years)	1151	12.08 (0.50)	11.99 (0.42)	0.002
**Anthropometric measurements**				
Weight (kg)	1151	49.21 (11.90)	48.66 (10.27)	0.403
Height (cm)	1151	155.13 (7.86)	155.41 (6.74)	0.514
BMI (kg/m^2^)	1151	20.28 (3.84)	20.06 (3.64)	0.318
**Blood pressure**				
SBP (mmHg)	1151	105.67 (11.09)	103.50 (10.24)	0.001
DBP (mmHg)	1151	60.81 (8.85)	62.63 (8.54)	< 0.001
**Biochemical analytes**				
BG (mg/dL)	1151	103.93 (11.75)	101.82 (11.71)	0.002
TC (mg/dL)	1151	151.59 (35.69)	154.59 (32.19)	0.133
HDL-c (mg /dL)	1149	62.92 (17.12)	62.85 (14.28)	0.934
LDL-c (mg/dL)	1083	75.80 (26.36)	77.43 (24.77)	0.296
TG (mg/dL)	1150	75.01 (42.48)	81.06 (36.88)	0.010
**Smoking status, n (%)**				0.014
Never smoked	1057	546 (90)	511 (94)	
**Physical activity, n (%)**				
≥ 60 min/day MVPA	387	263 (57)	124 (23)	< 0.001
< 60 min/day MVPA	764	344 (43)	420 (77)	
**Dietary intake**				
Fruit and vegetables (servings /day)	1149	3.31 (1.96)	3.64 (2.27)	0.010
Whole grains (servings/day)	1149	0.31 (0.57)	0.33 (0.62)	0.604
Fish (servings/week)	1150	4.44 (4.63)	4.19 (2.82)	0.258
Sweet beverages (mL/week)	1150	694.77 (1844.77)	533.78 (1453.81)	0.099
**Sociodemographic factors**				
Parental education, n (%)				
Low/medium	217	110 (19)	107 (20)	0.573
Medium	445	228 (40)	217 (42)	
High	431	234 (41)	197 (38)	
**Household income, n (%)**				
Low	353	181 (32)	172 (34)	0.734
Medium	333	172 (31)	161 (32)	
High	380	205 (37)	175 (34)	
**Municipality, n (%)**				
Barcelona	813	429 (69)	394 (72)	0.206
Madrid	338	188 (31)	150 (28)	

Data are expressed as mean (SD) or frequency (percentage).

N, number; SD, standard deviation; BMI, body mass index; SBP, systolic blood pressure; DBP, diastolic blood pressure; BG, blood glucose; TC, total cholesterol; HDL-C, high-density lipoprotein cholesterol; LDL-C, low-density lipoprotein cholesterol; TG, triglycerides; MVPA, moderate-to-vigorous physical activity.

Statistical analyses were carried out using the t-test for continuous variables and the chi-square test for categorical variables. *p* value s refer to differences between gender and are considered statistically significant when < 0.05.

Regarding the median overall CVH scores, 31% (n = 353) of participants had poor CVH, 64% (n = 735) intermediate, and 5% ideal (n = 63). Among the health behaviours, ideal smoking status was reported by more than 90% of boys and girls. No participant had an ideal healthy diet score. The BMI of approximately two-thirds of boys and three-quarters of girls was ideal. Only 23% of girls achieved ideal levels of physical activity compared with 43% of boys. Concerning the CVH factors, ideal TC and BP were reported in more than 60% and 90% of boys and girls, respectively. Less than 40% of all participants had ideal BG (Figure S1).

The mean urinary TPE of the participants was 125.29 (77.17) mg GAE/g creatinine. Overall, the adolescents in the highest tertile (T3) of TPE had lower values of body weight, BMI, BG, TC, LDL-C, and TG (Table S1). Boys and girls in T3 had lower values of TC, whereas only boys had lower values of BG (Table S2) (Table S3). All participants and girls had a higher daily intake of fruit and vegetables in the lowest tertile (T1) of TPE.

### TPE and overall CVH score

Results for the association between CVH score and TPE are presented in Table 3. In all regression models, higher levels of TPE were positively associated with higher CVH scores in all participants, and significant gender interaction was found. The gender-stratified analysis showed that only in boys were TPE tertiles directly associated with CVH scores in all the regression models.

**Table 3:** Association between CVH score and tertiles of TPE.

Overall CVH score	Models	n	TPE						
			T1	T2		T3		AIC	*p*-trend
				(B, 95% CI)	*p* value	(B, 95% CI)	*p* value		
All participants	Model 1	1151		0.21 (− 0.02; 0.43)	0.070	0.25 (0.12; 0.38)	< 0.001	3336.26	0.031
	Model 2	1144	Reference	0.17 (− 0.06; 0.40)	0.140	0.19 (0.14; 0.25)	< 0.001	3230.34	0.028
	Model 3	1050		0.11 (− 0.12; 0.34)	0.329	0.13 (0.10; 0.15)	< 0.001	2953.36	0.003
Boys	Model 1	607		0.15 (− 0.03; 0.32)	0.095	0.20 (0.12; 0.29)	< 0.001	1813.87	< 0.001
	Model 2	604	Reference	0.13 (− 0.08; 0.34)	0.232	0.18 (0.17; 0.19)	< 0.001	1765.86	< 0.001
	Model 3	551		0.08 (− 0.14; 0.31)	0.452	0.13 (0.12; 0.14)	< 0.001	1600.82	< 0.001
Girls	Model 1	544		0.09 (− 0.17; 0.35)	0.506	0.12 (− 0.08; 0.32)	0.235	1523.74	0.235
	Model 2	540	Reference	0.14 (− 0.07; 0.35)	0.182	0.12 (− 0.07; 0.30)	0.210	1461.21	0.210
	Model 3	499		0.12 (− 0.04; 0.27)	0.134	0.09 (− 0.07; 0.25)	0.261	1359.07	0.261

Multilevel mixed-effect linear regression models were used to evaluate the relationship between tertiles of TPE and overall CVH score (continuous). Model 1: adjusted by gender (also interaction). Model 2: adjusted as in model 1 plus age, Tanner stage, fasting, and triglycerides. Model 3: adjusted as in model 2 plus parental education and household income. Municipalities and schools were included as a random effect. *p* value T3 vs. T1 of TPE, and p-trend of tertiles of TPE < 0.05 are statistically significant.

AIC, Akaike information criteria; B, non-standardized coefficient; CI, confidence interval; CVH, cardiovascular health; TPE, total polyphenol excretion expressed as mg gallic acid equivalent (GAE)/g creatinine; T1, first tertile of TPE (< 85.8 mg GAE/g creatinine); T2, second tertile of TPE (85.8–140.5 mg GAE/g creatinine); T3, third tertile of TPE (> 140.5 mg GAE/g creatinine).

### TPE and each CVH metric

The distribution of CVH score and individual CVH metrics according to tertiles of TPE is shown in Figure S2 and Table S4, respectively. Lower TPE levels were more frequent in adolescents with poor CVH (Figure S2). Regarding ideal CVH metrics, a major difference between tertiles of TPE was only found for TC (*p* value = 0.003, Table S4).

The results of logistic regressions between TPE and individual CVH metrics are shown in Table 4. A total of 1050 participants were included in the analysis after the adjustment of covariates. The adjusted analysis revealed that the highest TPE tertile was associated with increased odds of ideal smoking status, ideal physical activity, and ideal TC, whereas it was less associated with ideal BMI compared to the lowest tertile of TPE. All participants followed a non-ideal healthy diet and logistic regression analysis could not be applied.

**Table 4:** Association between CVH metrics and tertiles of TPE.

CVH metrics	TPE All participants (n = 1050)					
	T1	T2		T3		*p*-trend
		(OR, 95% CI)	*p* value	(OR, 95% CI)	*p* value	
**Health behaviours**						
Ideal SS	Reference	1.16 (0.99; 1.35)	0.070	1.44 (1.10; 1.87)	0.007	0.231
Ideal BMI percentile	Reference	1.01 (0.92; 1.10)	0.872	0.87 (0.80; 0.93)	< 0.001	0.696
Ideal PA level	Reference	1.19 (0.57; 2.51)	0.642	1.12 (1.02; 1.23)	0.022	0.999
**Health factors**						
Ideal TC	Reference	1.21 (0.75; 1.94)	0.433	1.78 (1.16; 2.73)	0.009	0.009
Ideal BG	Reference	1.22 (0.82; 1.81)	0.321	1.10 (0.84, 1.46)	0.486	0.850
Ideal BP	Reference	1.25 (0.69; 2.27)	0.467	1.09 (0.68; 1.76)	0.709	0.419

Multilevel mixed-effect logistic regression was used to evaluate the relationship between tertiles of TPE and overall CVH metrics, adjusted by gender (also interaction), age, Tanner stage, fasting, triglycerides, parent education, and household income.

OR, odds ratio; CI, confidence interval; CVH, cardiovascular health; TPE, total polyphenol excretion expressed as mg gallic acid equivalent (GAE)/g creatinine; T1, first tertile of TPE (< 85.8 mg GAE/g creatinine); T2, second tertile of TPE (85.8–140.5 mg GAE/g creatinine); T3, third tertile of TPE (> 140.5 mg GAE/g creatinine); SS, smoking status; BMI, body mass index; PA, physical activity; TC, total cholesterol; BP, blood pressure; BG, blood glucose.

**Figure 1:**
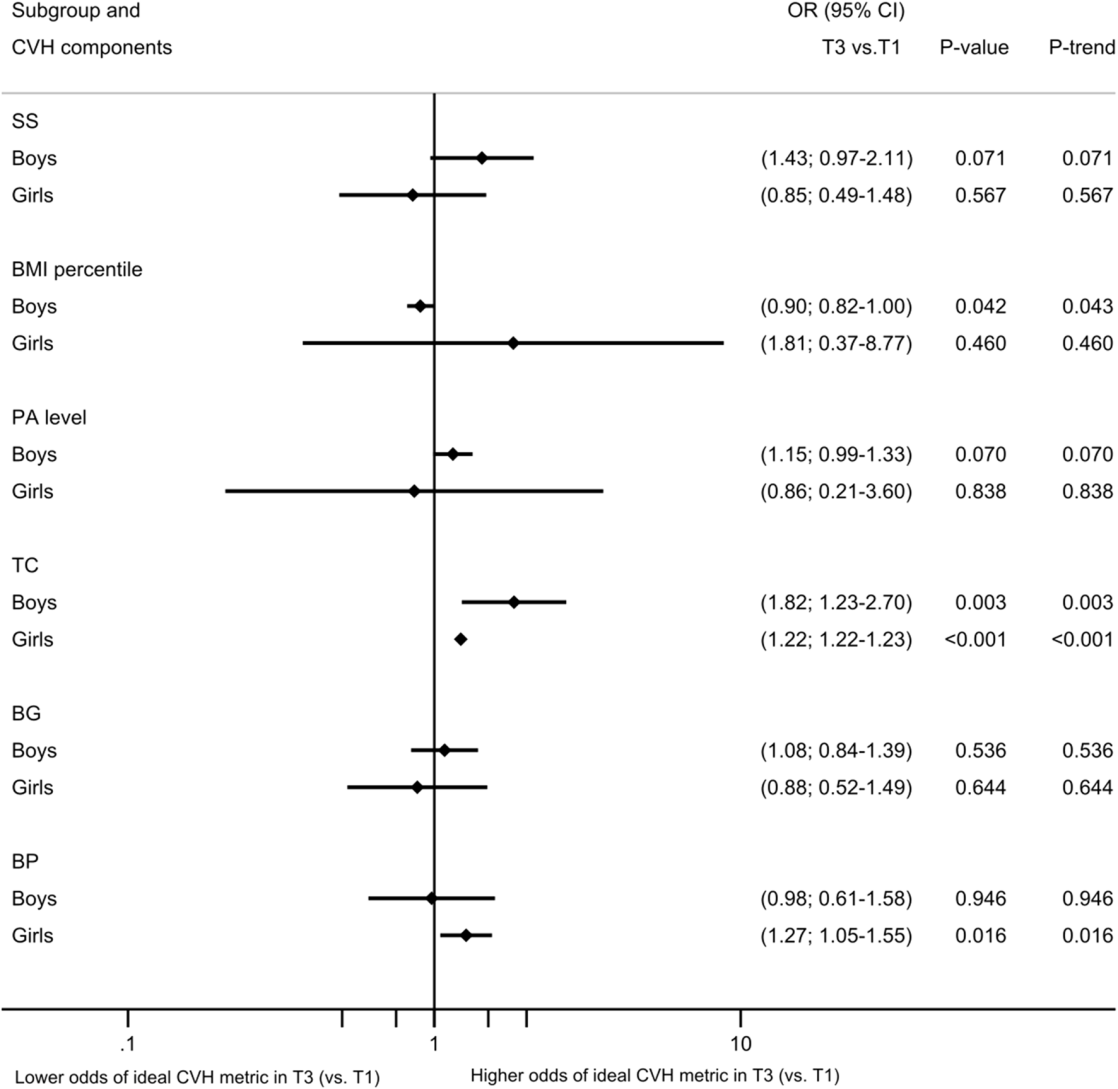
Association between ideal CVH metrics and tertiles of TPE by gender. OR, odds ratio; CI, confidence interval; CVH, cardiovascular health; TPE, total polyphenol excretion expressed as mg gallic acid equivalent (GAE)/g creatinine; T1, first tertile of TPE (< 85.8 mg GAE/g creatinine); T2, second tertile of TPE (85.8–140.5 mg GAE/g creatinine); T3, third tertile of TPE (> 140.5 mg GAE/g creatinine); SS, smoking status; BMI, body mass index; PA, physical activity; TC, total cholesterol; BP, blood pressure; BG, blood glucose. Multilevel mixed-effect logistic regression was used to evaluate the relationship between tertiles of TPE and each ideal CVH metric. All the analysis was adjusted by age, Tanner stage, fasting, TG, parent education, and household income. Municipalities and schools were included as a random effect. *p* value T3 vs. T1 of TPE, p-trend of tertiles of TPE, and p-interaction < 0.05 are statistically significant. All participants presented a non-ideal healthy diet and logistic regression analysis could not be applied.

Gender-specific analysis revealed that in boys, T3 was less associated with an ideal BMI compared to T1 and directly associated with TC, whereas in girls, the odds of having ideal TC (OR:1.22, 95% CI:1.22; 1.23, *p* value < 0.001) and ideal BP (OR:1.27, 95% CI:1.05; 1.55, *p* value = 0.016) were higher in T3 compared to T1 (Fig. 1). As all participants followed a non-ideal healthy diet, logistic regression analysis could not be applied.

## Discussion

In this cross-sectional study, the relationship between urinary TPE and CVH was investigated in 1151 adolescents aged 11 to 14 years. The findings indicate that higher TPE in urine is positively associated with a higher (healthier) CVH score, mainly due to specific CVH metrics, namely ideal smoking status, physical activity and total cholesterol, with some gender differences observed.

### Prevalence of ideal overall CVH and individual metrics

CVH metrics in adolescence have been associated with subclinical CVD in adulthood, indicating the importance of maintaining an ideal CVH from an early stage in life to prevent future ill health^11,12^. A low prevalence of ideal CVH (5%) was observed in the adolescent participants, due above all to low levels of physical activity and healthy diet, results similar to those reported by Fernandez-Jimenez et al. in a previous study of the same cohort, as well as in other studies in adolescents^25,26^.

### Polyphenols and ideal cardiovascular health

The mean urinary TPE of 125.3 (77.2) mg GAE/g creatinine was higher to the results of Hussein et al*.,* who reported an average TPE of 89.5 (8.4) mg GAE/g creatinine in 49 Egyptian children aged 7–14 years, using the same Folin–Ciocalteu method^27^. Concerning the association between urinary TPE and the overall CVH score, the highest tertile of TPE was correlated with a higher CVH score in all regression models.

Behaviours such as smoking, insufficient physical activity, and an unhealthy diet during adolescence are reported to increase the risk of subsequently developing CVD^1,11^. Although health behaviours are independently related to increased cardiovascular risk factors, clustering analysis of multiple lifestyle factors reveals a synergetic effect. Santaliestra-Pasías et al*.* suggested that the joint influence of low fruit and vegetable intake, a highly sedentary lifestyle, and low physical activity levels are related to excess body fat in European children aged 2–9 years^28^. The Mediterranean diet, characterized by the daily consumption of polyphenol-rich foods, is directly associated with higher TPE^9^, but in addition, adherence to the Mediterranean diet is linked with cultural behaviours that facilitate a healthy lifestyle^29^. In the present study, higher TPE was correlated with a greater likelihood of ideal smoking status and physical activity. Olmedo-Requena et al*.* reported that lower adherence to the MD was positively related with smoking habits and a sedentary lifestyle in young Spanish women^30^.

Higher urinary TPE was associated with lower odds of having an ideal BMI, in contrast with a previous study with the same cohort, where higher TPE was related to a lower BMI z-score (continuous) in boys^10^. In the present study, the ideal BMI was analysed as a dichotomic variable, above or equal to the 85th percentile and under the 85th percentile, the latter therefore including participants below the 5th percentile of BMI (underweight, n = 31). However, when the analysis was carried out with and without this underweight group, the results were similar. Also, linear regression between the BMI z-score (continuous) and tertiles of TPE was analysed, and a negative association was found between the highest tertile and the BMI z-score (B = -0.17, *p* value < 0.001). Wisnuwardani et al*.* showed that a higher intake of polyphenols, mainly flavonoids, was related with a lower BMI z-score in European adolescents aged 12.5 to 17.5 years^31^.

Regarding health factors, higher TPE was associated with a greater probability of having ideal TC in both genders, and ideal BP in girls. In a meta-analysis of five prospective cohort studies, Godos et al*.* reported that a higher intake of anthocyanins was associated with a reduced hypertension risk^32^. A reduction of TC in overweight/obese subjects due to the intake of products rich in ellagitannins and anthocyanins has been observed in several clinical trials^33^.

### Gender differences in the relation of polyphenols with cardiovascular health

In this study gender differences were observed in the associations between TPE levels and overall CVH and individual metrics. The stimulatory effect of polyphenols on the growth of beneficial microbiota and inhibition of pathogenic strains can vary according to gender and BMI. Studies suggest that women harbour a higher portion of *Firmicutes/Bacteroidetes*, whereas men with a lower BMI have a lower *Firmicutes/Bacteroidetes* ratio and therefore less risk of dysbiosis^34^. Additionally, women have been observed to have a higher number of *lactobacilli,* which could also generate gender-differential effects on BP^35^. The influence of polyphenols on the microbiota was not analysed here.

The changes in metabolic health status induced by increased sexual hormone secretion after puberty onset, including fat mass distribution, and levels of TC, leptin, and adiponectin, also differ between boys and girls^36,37^. Moreover, the secretion of androgens and oestrogens can be affected by phenolic phytoestrogens^38^. Accordingly, the Tanner stage was included as an adjustment variable in our study.

Gender differences have also been described in fatty acid metabolism, with women being more sensitive to the antilipolytic effects of insulin than men, resulting in a greater release of free fatty acids that contribute to the production of TG and TC^39^. This could explain the higher triglyceride levels found in girls, although TC levels did not differ from those of boys. Other gender-specific mechanisms related to CVH and TPE need to be identified in further research.

### Possible mechanisms for the effect of polyphenols on cardiovascular health

Polyphenols are associated with a protective effect against CVD^40^. Clinical trials have demonstrated that these plant secondary metabolites have a therapeutic role in vascular disorders, inflammation processes, glucose metabolism, dyslipidemia, hypertension, and oxidative stress^5–7^. The mechanisms underlying their biological effects involve the initiation of cell signalling responses and their interaction with both extracellular and intracellular receptors^41^.

By altering lipid metabolism and inhibiting the oxidation of LDL-C, polyphenols can reduce atherosclerotic lesions and platelet aggregation, as well as ameliorate endothelial function, resulting in lower BP^42^. Catechins are reported to activate nitric oxide synthase, thus modulating flow-mediated dilation and the vasodilation of endothelial cells, which reduces BP^7,43^. Flavonoids from tea, cocoa, and apples have been associated with lower levels of TC, LDL-C, and an increase in HDL-C^6^. The effects of polyphenols on metabolic pathways related to TC and BP, both CVH risk factors, could explain the results obtained in this study, in which polyphenol intake, measured by polyphenol excretion in urine, was seen to have a positive impact on CVH metrics. A higher consumption of polyphenol-rich foods was also associated with other aspects of a healthy lifestyle, as participants with higher TPE were more likely to have an ideal smoking status and physical activity level. Inter-individual variability, such as gender, also played a role.

### Strengths and limitations

The main strength of this study is the large sample size (n = 1151), and the use of standardized procedures. Moreover, the stratified analysis according to boys and girls increases the generality of the results. To the best of our knowledge, no studies about the association between polyphenol intake and ideal CVH in adolescents have been published to date.

This study has limitations that should be considered when interpreting the results. The cross-sectional design does not allow the identification of causal relationships between exposure and outcomes. The use of self-reported questionnaires risks biased reporting of diets, physical activity, and smoking status. As participants did not use accelerometers in water sports or during sports championships, the physical activity levels could have been underestimated. Given that fasting can alter BG concentrations, it was included as an adjustment variable in the analysis, even though 3% (n = 29) of the participants were not fasting. Another limitation is the issue of residual confounding due to the use of categorical variables.

## Conclusion

The results of the present study suggest that higher concentrations of polyphenols excreted in urine are associated with a more favourable CVH score in adolescents, mainly explained by the metrics of smoking status, physical activity, and TC. Gender differences were observed in the results; in boys, a higher TPE was associated with a better overall CVH score and ideal TC, and in girls with higher odds of having ideal TC and BP. The important finding of this study indicates the need to conduct similar studies in other European countries and worldwide. Additionally, longitudinal studies and randomized trials are needed to confirm the relationship of polyphenols with CVH and evaluate their effectiveness in preventing CVD.

## Supplementary Information

Supplementary Information.

## Data Availability

The datasets generated during and/or analysed during the current study are not publicly available due to there are restrictions on the availability of the data for the SI! Program study. Requestor wishing to access the database used in this study can make a request to the Steering Committee (SC) chair: gsantos@fundacionshe.org, rodrigo.fernandez@cnic.es, juanmiguel.fernandez@cnic.es, RESTRUCH@clinic.cat, lamuela@ub.edu, bibanez@cnic.es, vfuster@cnic.es. For the present study, the database was requested from the SC on 4th June 2021.

## Abbreviations

BG: Blood glucose
BMI: Body mass index
BP: Blood pressure
CDC: Center for Disease Control
CEHQ: Children’s Eating Habit Questionnaire
CVH: Cardiovascular health
CVD: Cardiovascular disease
DBP: Diastolic blood pressure
FFQ: Food frequency questionnaire
GA: Gallic acid
HDL-C: High-density lipoprotein cholesterol
LDL-C: Low-density lipoprotein cholesterol
MVPA: Moderate-to-vigorous physical activity
OR: Odds ratio
SBP: Systolic blood pressure
TC: Total cholesterol
TG: Triglycerides
TPE: Total polyphenol excretion
