# Supplementary material for: Total urinary polyphenols and ideal cardiovascular health metrics in Spanish adolescents enrolled in the SI Program: a cross-sectional study
Source: Sci Rep. 2022 Sep 14;12:15468. doi: 10.1038/s41598-022-19684-6 (PMC9475038; doi:10.1038/s41598-022-19684-6)
Supplement: Supplementary file 1 — Supplementary Information. [file 41598_2022_19684_MOESM1_ESM.pdf]

Total urinary polyphenols and ideal cardiovascular health metrics in Spanish adolescents enrolled in the SI Program: a cross-sectional study

Emily P. Laveriano-Santos[a1,2], Camila Arancibia-Riveros[a1], Isabella Parilli-Moser[1,2], Sonia L. Ramírez-Garza[1], Anna Tresserra-Rimbau[1,2*], Ana María Ruiz-León[2,3,4], Ramón Estruch[2,3], Patricia Bodega[5,6], Mercedes de Miguel[5,6], Amaya de Cos-Gandoy[5,6], Vanesa Carral[5,6], Gloria Santos-Beneit[5,7], Juan M. Fernández-Alvira[6], Rodrigo Fernández-Jiménez[6,8,9], and Rosa M. Lamuela-Raventós[1,2*]

[1] Department of Nutrition, Food Science and Gastronomy, School of Pharmacy and Food Sciences XIA, Institute of Nutrition and Food Safety (INSA-UB), University of Barcelona, 08921 Santa Coloma de Gramenet, Spain.

[2] Consorcio CIBER, M.P. Fisiopatología de la Obesidad y Nutrición (CIBERObn), Instituto de Salud Carlos III (ISCIII), 28029 Madrid, Spain.

[3] Department of Internal Medicine, Hospital Clínic, Institutd'Investigacions Biomèdiques August Pi I Sunyer (IDIBAPS), University of Barcelona, 08036 Barcelona, Spain.

[4] Mediterranean Diet Foundation, 08021 Barcelona, Spain.

[5] Foundation for Science, Health and Education (SHE), 08008 Barcelona, Spain.

[6] Centro Nacional de Investigaciones Cardiovasculares (CNIC), 28029 Madrid, Spain.

[7] The Zena and Michael A. Wiener Cardiovascular Institute, Icahn School of Medicine at Mount Sinai, New York, NY 10029, USA

[8] CIBER de Enfermedades Cardiovasculares (CIBERCV), 28029 Madrid, Spain.

[9] Hospital Universitario Clínico San Carlos, 28040 Madrid, Spain.

[*]Correspondence : lamuela@ub.edu (Rosa M. Lamuela-Raventós), annatresserra@ub.edu (Anna Tresserra-Rimbau)

[a] Equally Contributing Authors

Online Supplementary Material

Supplementary tables

Table S1. Baseline characteristics of the SI! Program cohort at baseline by tertiles of TPE.

	N	Missing N (%)	T1 (n=384)	T2 (n=384)	T3 (n=383)	*P-value*
Girls	544		187 (49)	187 (49)	170 (44)	0.386
Age (y)	1151	0 (0)	12.03 (0.48)	12.05 (0.45)	12.02 (0.47)	0.638
Anthropometric measurements						
Weight (kg)	1151	0 (0)	49.95 (12.00)	48.98 (10.27)	47.92 (11.07)	0.043
Height (cm)	1151	0 (0)	155.17 (7.34)	155.47 (7.12)	155.14 (7.61)	0.794
BMI (kg/m^2)	1151	0 (0)	20.61 (4.11)	20.15 (3.46)	19.78 (3.61)	0.008
Blood pressure						
SBP (mmHg)	1151	0 (0)	105.34 (10.66)	103.89 (10.86)	104.69 (10.70)	0.173
DBP (mmHg)	1151	0 (0)	61.89 (9.12)	60.92 (8.64)	62.20 (8.46)	0.108
Biochemical analyses						
BG (mg/dL)	1151	0 (0)	103.99 (11.93)	103.16 (12.19)	101.64 (11.09)	0.020
TC (mg/dL)	1151	0 (0)	155.69 (32.33)	156.61 (37.05)	146.70 (31.87)	<0.001
HDL-C (mg /dL)	1149	2 (0)	63.39 (15.71)	63.95 (17.04)	61.31 (14.56)	0.052
LDL-C (mg/dL)	1083	68 (6)	77.63 (24.52)	78.55 (27.79)	73.52 (24.18)	0.020
TG (mg/dL)	1150	1 (0)	79.91 (44.80)	80.00 (38.06)	73.68 (36.48)	0.043
Smoking status, n(%)						0.866
Never smoked	1057	94 (8)	352 (92)	351 (91)	354 (92)	
Physical activity, n(%)	1151	0 (0)				0.683
≥60 min/day MVPA	387		122 (32)	131 (34)	134 (35)	
<60 min/day MVPA	764		262 (68)	253 (66)	249 (65)	
Dietary intake						
Fruit and vegetables (servings /day)	1149	2 (0)	3.69 (2.37)	3.33 (2.06)	3.38 (1.87)	0.033
Whole grains (servings /day)	1149	2 (0)	0.34 (0.69)	0.30 (0.53)	0.32 (0.54)	0.641
Fish (servings /week)	1150	1 (0)	4.48 (4.07)	4.32 (3.69)	4.15 (3.87)	0.499
Sweet beverages (mL/week)	1150	1 (0)	680.48 (1765.85)	552.45 (1483.01)	653.17 (1752.67)	0.376
Sociodemographic factors	1093	58 (5)				
Parental education, n(%)						0.866
Low	217		77 (21)	75 (21)	65 (18)	
Medium	445		147 (40)	148 (41)	150 (41)	
High	431		142 (39)	142 (38)	147 (41)	
Household income, n(%)	1066	85 (7)				0.444
Low	353		124 (35)	125 (34)	104 (30)	
Medium	333		113 (32)	112 (31)	108 (31)	
High	380		116 (33)	128 (35)	136 (39)	

Municipality, n(%)	1151	0 (0)				
Barcelona	813		283 (74)	284 (74)	246 (64)	0.003
Madrid	338		101 (26)	100 (26)	137 (36)	

Data are expressed as the mean (SD) or as percentage. Abbreviations: N number; SD standard deviation, TPE total polyphenol excretion expressed as mg gallic acid equivalent (GAE)/g creatinine, T1 first tertile of TPE (< 85.8mg GAE/g creatinine), T2 second tertile of TPE (85.8-140.5 mg GAE/g creatinine), T3 third tertile of TPE (> 140.5 mg GAE/g creatinine), BMI body mass index, SBP systolic blood pressure, DBP diastolic blood pressure, BG blood glucose, TC total cholesterol, HDL-C high-density lipoprotein cholesterol, LDL-C low-density lipoprotein cholesterol, TG triglycerides, MVPA moderate-to-vigorous physical activity. Statistical analyses were undertaken using one-way ANOVA for continuous variables and the chi-square test for categorical variables. P-values refer to differences between tertiles of TPE and are considered statistically significant when < 0.05.

Table S2. Baseline characteristics of the SI! Program cohort at baseline by tertiles of TPE in boys

	N	T1 (n=197)	T2 (n=197)	T3 (n=213)	*P-value*
Age (y)	607	12.09 (0.54)	12.09 (0.44)	12.05 (0.52)	0.647
**Anthropometric measurements**					
Weight (kg)	607	50.27 (12.49)	48.86 (11.05)	48.55 (12.10)	0.301
Height (kg)	607	155.19 (7.81)	155.34 (7.52)	154.89 (8.25)	0.842
BMI (kg/m^2)	607	20.72 (4.13)	20.10 (3.57)	20.06 (3.80)	0.160
**Blood pressure**					
SBP (mmHg)	607	106.61 (11.07)	105.13 (11.22)	105.29 (11.00)	0.342
DBP (mmHg)	607	61.29 (9.08)	59.81 (8.81)	61.28 (8.64)	0.159
**Biochemical analytes**					
BG (mg/dL)	607	105.76 (11.85)	103.63 (12.36)	102.51 (10.90)	0.018
TC (mg/dL)	607	154.08 (33.43)	155.86 (41.11)	145.33 (31.37)	0.006
HDL-C (mg /dL)	605	62.42 (16.61)	65.28 (18.83)	61.22 (15.71)	0.050
LDL-C (mg/dL)	563	78.26 (25.32)	76.68 (30.18)	72.70 (23.13)	0.104
TG (mg/dL)	606	79.71 (53.56)	75.76 (39.13)	69.93 (32.24)	0.064
**Smoking status**, n(%)					
Never smoked	546	175 (89)	176 (89)	195 (92)	0.620
**Physical activity**, n(%)					0.426
≥60 min/day MVPA	263	78 (40)	90 (46)	95 (45)	
<60 min/day MVPA	344	119 (60)	107 (54)	118 (55)	
**Dietary** intake					
Fruit and vegetables (servings /day)	607	3.38 (1.97)	3.15 (1.96)	3.40 (1.93)	0.372
Whole grains (servings /day)	605	0.31 (0.55)	0.31 (0.54)	0.31 (0.61)	0.985
Fish (servings /week)	606	4.60 (5.05)	4.50 (4.01)	4.24 (4.76)	0.718
Sweet beverages (ml /week)	606	808.80 (1963.97)	532.53 (1536.93)	739.88 (1986.25)	0.302
**Sociodemographic factors**					
**Parental education**, n(%)					
Low	110	45 (24)	38 (21)	27 (13)	0.040
Medium	228	62 (33)	74 (40)	92 (46)	
High	234	79 (43)	73 (39)	82 (41)	
**Household income**, n(%)					
Low	181	63 (35)	63 (34)	55 (28)	0.287
Medium	172	59 (33)	56 (31)	57 (29)	

High	205	58 (32)	64 (35)	83 (43)	
**Municipality**, n(%)					
Barcelona	419	138 (70)	142 (72)	139 (65)	0.306
Madrid	188	59 (30)	55 (28)	74 (35)	

Data are expressed as the mean (SD) or as percentage. Abbreviations: N number, SD standard deviation, TPE total polyphenol excretion expressed as mg gallic acid equivalent (GAE)/g creatinine, BMI body mass index, SBP systolic blood pressure, DBP diastolic blood pressure, BG blood glucose, TC total cholesterol, HDL-C high-density lipoprotein cholesterol, LDL-C low-density lipoprotein cholesterol, TG triglycerides, MVPA moderate-to-vigorous physical activity. Statistical analyses were carried out using one-way ANOVA for continuous variables and the chi-square test for categorical variables. P-values refer to differences between tertiles of TPE and are considered statistically significant when < 0.05.

Table S3. Baseline characteristics of the SI! Program cohort at baseline by tertiles of TPE in girls

	N	T1 (n=187)	T2 (n=187)	T3 (n=170)	*P-value*
Age (y)	544	11.97 (0.41)	12.02 (0.45)	11.99 (0.39)	0.602
**Anthropometric measurements**					
Weight (kg)	544	49.60 (11.49)	49.10 (9.41)	47.15 (9.64)	0.060
Height (kg)	544	155.16 (6.84)	155.61 (6.69)	155.46 (6.73)	0.806
BMI (kg/m^2)	607	20.50 (4.09)	20.20 (3.35)	19.42 (3.33)	0.015
**Blood pressure**					
SBP (mmHg)	544	104.01 (10.07)	102.59 (10.34)	103.95 (10.30)	0.326
DBP (mmHg)	544	62.51 (9.14)	62.09 (8.31)	63.35 (8.10)	0.366
**Biochemical analytes**					
BG (mg/dL)	544	102.13 (11.76)	102.66 (12.02)	100.56 (11.26)	0.218
TC (mg/dL)	544	157.40 (31.13)	157.40 (32.32)	148.42 (32.51)	0.010
HDL-C (mg /dL)	544	64.41 (14.69)	62.57 (14.86)	61.44 (13.04)	0.138
LDL-C (mg/dL)	520	77.00 (23.75)	80.50 (24.99)	74.53 (25.46)	0.084
TG (mg/dL)	544	80.11 (33.33)	84.47 (36.47)	78.35 (40.78)	0.267
**Smoking status**, n(%)					
Never smoked	511	177 (95)	175 (94)	159 (94)	0.879
**Physical activity**, n(%)					
≥60 min/day MVPA	124	44 (26)	41 (22)	39 (23)	0.932
<60 min/day MVPA	420	143 (74)	146 (78)	131 (77)	
**Dietary** intake					
Fruit and vegetables (servings /day)	542	4.02 (2.69)	3.51 (2.15)	3.36 (1.80)	0.014
Whole grains (servings /day)	544	0.37 (0.81)	0.29 (0.52)	0.33 (0.44)	0.467
Fish (servings /week)	544	4.36 (2.68)	4.14 (3.33)	4.05 (2.32)	0.548
Sweet beverages (mL /week)	544	545.97(1526.07)	511.83 (1428.06)	544.53 (1404.67)	0.968
**Sociodemographic factors**					
**Parental education**, n(%)					
Low	107	32 (18)	37 (21)	38 (24)	0.325
Medium	217	85 (47)	74 (41)	58 (36)	
High	197	63 (35)	69 (38)	65 (40)	
**Household income**, n(%)					
Low	172	61 (35)	62 (34)	49 (32)	0.972

Medium	161	54 (31)	56 (31)	51 (33)	
High	175	58 (33)	64 (35)	53 (35)	
**Municipality**, n(%)					
Barcelona	394	145 (78)	142 (76)	107 (63)	0.004
Madrid	150	42 (22)	45 (24)	63 (37)	

Data are expressed as the mean (SD) or as percentage. Abbreviations: N number; SD standard deviation, TPE total polyphenol excretion expressed as mg gallic acid equivalent (GAE)/g creatinine, BMI body mass index, SBP systolic blood pressure, DBP diastolic blood pressure, BG blood glucose, TC total cholesterol, HDL-C high-density lipoprotein cholesterol, LDL-C low-density lipoprotein cholesterol, TG triglycerides, MVPA moderate-to-vigorous physical activity. Statistical analyses were carried out using one-way ANOVA for continuous variables and the chi-square test for categorical variables. P-values refer to differences between tertiles of TPE and are considered statistically significant when < 0.05.

Table S4. CVH metrics by tertiles of TPE

	Total (n=1151)	T1 (n=384))	T2 (n=384)	T3 (n=383)	*P-value*
Overall CVH, n (%)					
Ideal	63 (5)	21 (6)	15 (4)	27 (7)	<0.001
Intermediate	735 (64)	216 (56)	262 (68)	257 (67)	
Poor	353 (31)	147 (38)	107 (28)	99 (26)	
SS, n (%)					
Ideal	1057 (92)	352 (92)	351 (91)	354 (92)	0.866
Non-ideal	94 (8)	32 (8)	33 (9)	29 (8)	
BMI percentile, n (%)					
Ideal	841 (73)	267 (70)	289 (75)	285 (74)	0.155
Non-ideal	310 (27)	117 (30)	95 (25)	98 (26)	
PA level, n (%)					
Ideal	387 (34)	122 (32)	131 (34)	134 (35)	0.622
Non-ideal	764 (66)	262 (68)	253 (66)	249 (65)	
HDS, n (%)					
Non-ideal	1151 (100)	384 (100)	384 (100)	383 (100)	
TC, n (%)					
Ideal	809 (70)	253 (66)	263 (68)	293 (76)	0.004
Non-ideal	342 (30)	131 (34)	121 (32)	90 (24)	
BG, n (%)					
Ideal	429 (37)	133 (35)	146 (38)	150 (39)	0.402
Non-ideal	722 (63)	251 (65)	238 (62)	233 (61)	
BP, n (%)					
Ideal	1044 (91)	342 (89)	351 (91)	351 (92)	0.396
Non-ideal	107 (9)	42 (11)	33 (9)	32 (8)	

Data are expressed as percentages. Abbreviations: N number; CVH cardiovascular health, TPE total polyphenol excretion expressed as mg gallic acid equivalent (GAE)/g creatinine, T1 first tertile of TPE (< 85.8mg GAE/g creatinine), T2 second tertile of TPE (85.8-140.5 mg GAE/g creatinine), T3 third tertile of TPE (> 140.5 mg GAE/g creatinine), SS smoking status, BMI body mass index, PA physical activity, HDS healthy diet score, TC total cholesterol, BP blood pressure, BG blood glucose. Statistical analyses were undertaken using the chi-square test. P-values refer to differences between tertiles of TPE and are considered statistically significant when < 0.05.

Figures

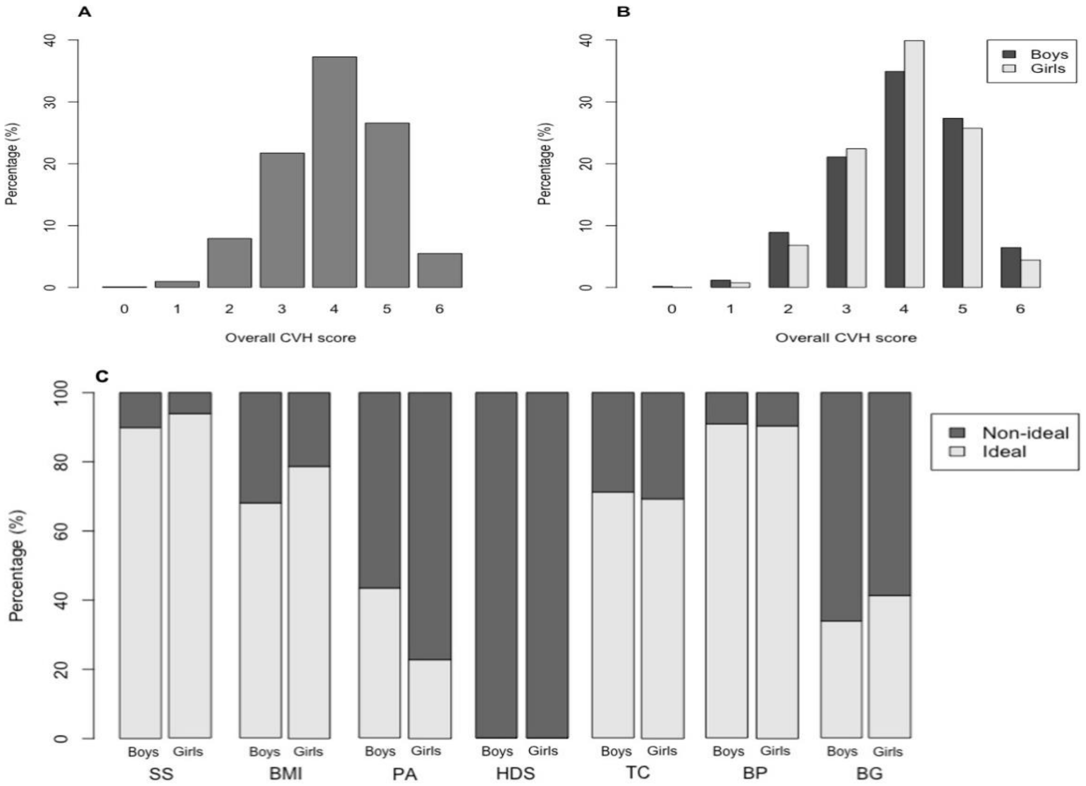

Figure S1. Prevalence of Cardiovascular Health (CVH) score, CVH behaviors and factors.

A: Ideal CVH score in all participants. B: Ideal CVH score by gender. C: Ideal CVH behaviors and factors. SS smoking status, BMI body mass index, PA physical activity, HDS healthy diet score, TC total cholesterol, BP blood pressure, BG blood glucose.

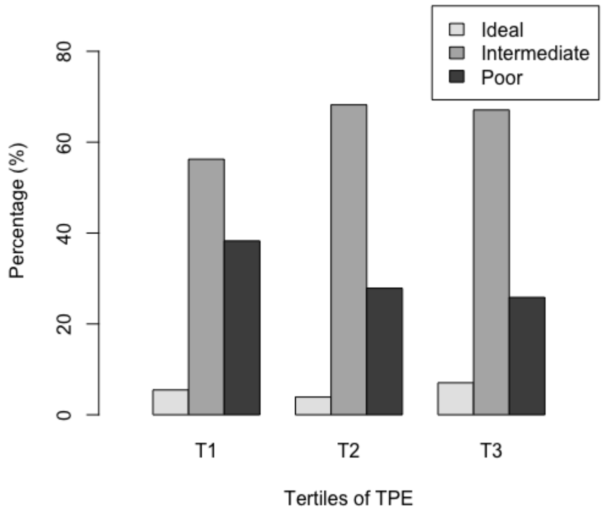

Figure S2. Cardiovascular Health (CVH) categories by tertiles of TPE.

TPE total polyphenol excretion expressed as mg gallic acid equivalent (GAE)/g creatinine, T1 first tertile of TPE (< 85.8mg GAE/g creatinine), T2 second tertile of TPE (85.8-140.5 mg GAE/g creatinine), T3: third tertile of TPE (> 140.5 mg GAE/g creatinine).
